# The Potential of Frog Skin-Derived Peptides for Development into Therapeutically-Valuable Immunomodulatory Agents

**DOI:** 10.3390/molecules22122071

**Published:** 2017-12-13

**Authors:** Jelena M. Pantic, Ivan P. Jovanovic, Gordana D. Radosavljevic, Nebojsa N. Arsenijevic, J. Michael Conlon, Miodrag L. Lukic

**Affiliations:** 1 Center for Molecular Medicine and Stem Cell Research, Faculty of Medical Sciences, University of Kragujevac, 34000 Kragujevac, Serbia; ivanjovanovic77@gmail.com (I.P.J.); perun.gr@gmail.com (G.D.R.); arne@medf.kg.ac.rs (N.N.A.); 2 SAAD Centre for Pharmacy and Diabetes, School of Biomedical Sciences, University of Ulster, Cromore Road, Coleraine, Northern Ireland BT52 1SA, UK; jmconlon1@gmail.com

**Keywords:** frog skin peptides, host defence peptides, immunomodulatory, cytokine, antimicrobial

## Abstract

The aim of this article is to review the immunoregulatory actions of frog skin-derived peptides in order to assess their potential as candidates for immunomodulatory or anti-inflammatory therapy. Frog skin peptides with demonstrable immunomodulatory properties have been isolated from skin secretions of a range of species belonging to the families Alytidae, Ascaphidae, Discoglossidae, Leptodactylidae, Pipidae and Ranidae. Their effects upon production of inflammatory and immunoregulatory cytokines by target cells have been evaluated ex vivo and effects upon cytokine expression and immune cell activity have been studied in vivo by flow cytometry after injection into mice. The naturally-occurring peptides and/or their synthetic analogues show complex and variable actions on the production of proinflammatory (TNF-α, IL-1β, IL-12, IL-23, IL-8, IFN-γ and IL-17), pleiotropic (IL-4 and IL-6) and immunosuppressive (IL-10 and TGF-β) cytokines by peripheral and spleen cells, peritoneal cells and/or isolated macrophages. The effects of frenatin 2.1S include enhancement of the activation state and homing capacity of Th1-type lymphocytes and NK cells in the mouse peritoneal cavity, as well as the promotion of their tumoricidal capacities. Overall, the diverse effects of frog skin-derived peptides on the immune system indicate their potential for development into therapeutic agents.

## 1. Introduction

Antimicrobial peptides are an integral component of the system of innate immunity associated with all organisms and, in vertebrates, are abundantly produced in skin and mucosal epithelia and by some innate immune cells [1,2]. The primary role of such compounds is to create a chemical barrier as a protection from invading pathogens in the environment, and they may also act as a deterrent against ingestion by predators [3,4]. As well as their microbicidal actions, additional protective capacities of such molecules are now recognized, including their abilities to affect the host immune system [5,6,7]. In view of their multifunctional nature, these peptides are best regarded as host-defence peptides, rather than exclusively antimicrobial peptides, and they are generally active across species [8].

Peptides with diverse structural characteristics and biological effects are of particular importance in the host defence mechanisms of anurans (frogs and toads). To date, about 1000 such peptides, obtained primarily from frog species belonging to the Ascaphidae, Alytidae, Bombinatoridae, Hylidae, Hyperoliidae, Leptodactylidae, Myobatrachidae, Pipidae and Ranidae families, are included in Antimicrobial Peptide Database [9,10,11]. Numerous chemically-modified analogues of the frog peptides with improved pharmacological properties have been synthetized in order to increase their potential for therapeutic applications [12,13,14]. The frog host defence peptides are synthetized and stored in granular glands in the skin, often in very high concentrations, and are rapidly released following stress or tissue injury [15,16]. Naturally-occurring frog skin-derived peptides vary appreciably in their primary structure and lack any conserved domains responsible for their biological actions. However, the vast majority of frog skin peptides are cationic and contain a high proportion of hydrophobic amino acids. With a few exceptions, they lack a stable conformation in aqueous solutions, but adopt an amphipathic α-helical structure in the environment of a phospholipid vesicle or in a membrane-mimetic solvent such as 50% trifluoroethanol–water [9].

Frog skin host defence peptides have been shown to display a wide range of biological effects in mammals. They were first recognized not only for their wide ranging antimicrobial (antibacterial, antifungal, anti-protozoal and antiviral) effects, but also for their ability to permeabilize mammalian cells [11]. In the absence of recognized conserved domains, as well as specific receptors responsible for their antimicrobial and cytotoxic effects, the mechanism of action of the peptides generally involves direct disruption of the cell membrane [17,18]. The microbicidal actions of frog skin peptides against multidrug-resistant pathogens have attracted considerable attention, and numerous synthetic analogues with increased antimicrobial potency, decreased toxicity and longer half-lives in circulation have been evaluated [19]. The therapeutic potential of frog skin host defence peptides is not confined to their role as antimicrobial agents. Several compounds have been proposed as anti-cancer agents based on their selective cytotoxicity against various tumour cells [20]. Anti-cancer effects of the peptides arise from direct disruption of the tumour cell membrane, but may also involve the induction of apoptotic cell death or the inhibition of tumour angiogenesis [21,22]. Additionally, it has been suggested that some frog skin peptides have the potential for development into agents for type 2 diabetes therapy (reviewed in [23,24]). The peptides exhibit anti-diabetogenic effects in various in vitro and in vivo experimental models, including the ability to increase insulin secretion and insulin sensitivity, improved glucose tolerance, suppression of glucagon release, enhanced β-cell proliferation, protection against β-cell apoptosis, as well as improved lipid status.

Host defence peptides are involved in the stimulation of the protective immune response, while suppressing the harmful inflammatory response [5,6,7]. The organizations of amphibian and mammalian immune systems share numerous similarities in terms of the functions of both innate and adaptive immunity [25,26]. Genes encoding proinflammatory cytokines, such as interleukin (IL)-1, IL-6, tumour necrosis factor (TNF)-α and interferon (IFN)-γ, are present in the genome of *Xenopus tropicalis* (formerly *Silurana tropicalis*), and activation of skin macrophages in response to extracellular pathogens has been reported [25,27]. In addition, dermal peptides are important participants in epithelial tissue repair following injury in both Amphibia and mammals [28]. An early indication that frog skin host defence peptides might be involved in the control of immune response is related to their chemoattractant properties. It was observed that temporin A and related peptides enhance pertussis toxin-dependent influx of phagocytes, including macrophages, monocytes and neutrophils [29].

This article evaluates the immunomodulatory potential of a range of naturally-occurring frog skin peptides, as well as their synthetic analogues. Effects on the production of various cytokines of innate and/or adaptive immunity are determined using different immune cell cultures including mouse peritoneal cells, isolated macrophages and spleen cells and human peripheral mononuclear (PBM) cells, with and without concomitant stimulation with lipopolysaccharide (LPS) or concanavalin A (Con A). Assessment of immunomodulatory effects of frog skin peptides in vivo involves an examination of their abilities to promote immune cells’ influx and activation status in different strains of mice. The species from which frog skin immunomodulatory peptides have been isolated are summarized in Table 1, and the primary structures of the peptides are shown in Table 2.

## 2. Frog Skin Peptides with Predominantly Immunostimulatory Activity that Lack Antimicrobial Activity

Frenatin 2D, first isolated from norepinephrine-stimulated skin secretions of the Tyrrhenian painted frog *Discoglossus sardus* (Alytidae), did not show any microbicidal or haemolytic activity at concentrations of up to 300 μM [32]. At a concentration of 20 μg/mL, the peptide enhanced the release of proinflammatory cytokines TNF-α and IL-1β, but not IL-6, by mouse peritoneal macrophages. In addition, frenatin 2D stimulated the production of IL-12 in both LPS-stimulated and unstimulated murine peritoneal macrophages.

Plasticin-L1 is a glycine/leucine-rich peptide originating from norepinephrine-stimulated skin secretions of the South-American Santa Fe frog *Leptodactylus laticeps* (Leptodactylidae) [34]. Plasticin-L1 did not display antimicrobial activity consistent with its weak cationic nature and consequent low affinity for bacterial cell membranes. However, at a concentration of 20 μg/mL, the peptide enhanced the production of proinflammatory IL-1β, IL-12, IL-23 and TNF-α by peritoneal macrophages from both C57BL/6 and BALB/c mice. Plasticin-L1 increased the production of IL-6 by macrophages in the presence or absence of LPS, without a significant effect on the anti-inflammatory IL-10 [34].

## 3. Frog Skin Peptides with Predominantly Anti-Inflammatory Activity that Lack Antimicrobial Activity

The tigerinins are a family of small, cationic, cyclic peptides, some of which contain an α-amidated *C*-terminus. Tigerinin-1R from *Hoplobatrachus rugulosus* (Dicroglossidae) [43], tigerinin-1V from *Lithobates vaillanti* (Ranidae) [44] and tigerinin-1M from *Xenopus muelleri* (Pipidae) [45] did not exhibit any microbicidal or haemolytic actions at concentration up to 500 μg/mL [35]. The most striking immunomodulatory effect of the three tigerinins is a significant dose-dependent stimulation of anti-inflammatory IL-10 production by murine peritoneal macrophages and splenocytes, as well as by human peripheral blood mononuclear cells in both LPS-stimulated and unstimulated cells. In addition, the tigerinins (20 μg/mL) enhanced the release of IL-6 in LPS-stimulated macrophages from C57BL/6 mice, but only tigerinin-1V potentiated IL-6 production in LPS-stimulated macrophages from BALB/c mice. The effects on the production of proinflammatory IL-12 and IL-23 by macrophages from both mouse strains were less clearly defined or absent. In a population of mononuclear cells derived from mouse spleen, tigerinin-1M and -1V suppressed the production of IFN-γ, without a significant effect on IL-17 production [35]. A further four host-defence peptides belonging to the tigerinin family (tigerinin-1O, tigerinin-2O, tigerinin-3O and tigerinin-4O) were isolated from skin secretions of the African crowned bullfrog *Hoplobatrachus occipitalis* (Dicroglossidae) [36]. These tigerinins also lacked antimicrobial and haemolytic activities, but at a concentration of 20 μg/mL, significantly inhibited production of IFN-γ by peritoneal cells from C57BL/6 mice without affecting the production of IL-10 and IL-17. Tigerinin-2O and -4O inhibited IFN-γ production at concentrations as low as 1 μg/mL.

The skin secretions of toad *Rhinophrynus dorsalis* (Rhinophrynidae) are a source of the proline-arginine-rich peptides rhinophyrinin-33 and the truncated form rhinophyrinin-27, which adopt a poly-proline helical conformation rather than the more usual α-helix [37]. Although rhinophrynin-27 shows limited structural similarity with the porcine multifunctional peptide PR-39, it does not exhibit antimicrobial or cytotoxic effects. However, preliminary investigation of the immunomodulatory effects of rhinophrynin-27 indicates a dose-dependent suppression of the production of proinflammatory TNF-α by murine macrophages [38].

## 4. Frog Skin Peptides with Antimicrobial and Immunosuppressive Activities

The potential of analogues of frog skin peptides, selected for the increased potency against microrganisms and decreased cytotoxicity against mammalian cells, was evaluated as candidates for the topical treatment of acne vulgaris. [D4k]ascaphin-8 [46], [G4K]XT-7 [46], [T5k]temporin-DRa [12], brevinin-2GU and B2RP-ERa, derived from peptides originally isolated from *Ascaphus truei* [47], *Xenopus tropicalis* [48], *Rana draytonii* [49], *Hylarana guentheri* [50] and *Hylarana erythraea* [51], inhibited the growth of *Propionibacterium acnes*, the bacterium associated with infection of the pilosebaceous unit, but showed only weak haemolytic activity against human erythrocytes [30]. [D4k]ascaphin-8, [G4K]XT-7, brevinin-2GU and B2RP-ERa (1 and 20 μg/mL), as well as [T5k]temporin-DRa (20 μg/mL) significantly reduced the production of TNF-α from Con A-stimulated PBM cells. [D4k]ascaphin-8 and brevinin-2GU suppressed the production of IFN-γ from unstimulated PBM cells at concentrations of 1 and 20 μg/mL, while [T5k]temporin-DRa and B2RP-ERa enhanced the release of anti-inflammatory transforming growth factor (TGF)-β, IL-4 and IL-10 from both unstimulated and Con A-treated PBM cells.

Hymenochirin-1B was initially isolated from skin secretions of the Congo clawed frog *Hymenochirus boettgeri* (Pipidae) [52]. The naturally-occurring peptide was chemically modified by substitution of several amino acids with L- or D-lysine in order to increase cationicity, without affecting amphipathicity, with a view toward obtaining an analogue with increased microbicidal, but lower haemolytic activity. [E6k,D9k]hymenochirin-1B, as well as exhibiting potent microbicidal activity against a wide range of multidrug-resistant bacteria, enhanced the production of anti-inflammatory IL-4 and IL-10 by human PBM cells, while the effects on the production of proinflammatory TNF-α and IL-17 were not significant [39].

## 5. Frog Skin Peptides with Antimicrobial, Cytotoxic and Immunostimulatory Activities

Alyteserin-2 was first isolated from skin secretions of the midwife toad *Alytes obstetricans* (Alytidae) [53] and, in common with the majority of frog skin host defence derivatives, is positively charged and has the propensity to adopt an amphipathic α-helical conformation. Compared with the native peptide, the analogue [G11k,N15K]alyteserin-2a showed selective cytotoxicity for a range of human tumour cell lines, and the peptide (1 μg/mL) significantly inhibited the release of the immune-suppressive cytokines IL-10 and TGF-β from unstimulated and Con A-stimulated PBM cells [31].

Esculentin-2CHa was first isolated from skin of the Chiricahua leopard frog, *Lithobates chiricahuensis* (Ranidae) [54], and analogues of the peptide have been synthesized that show diverse biological activities depending on their cationicity, α-helicity, hydrophobicity and amphipathicity [42]. The naturally-occurring peptide shows potent growth inhibitory activity against a range of multidrug-resistant bacteria, as well as cytotoxic activity against human erythrocytes and human non-small cell lung adenocarcinoma A549 cells. Esculentin-2CHa significantly stimulates the release of the anti-inflammatory cytokine IL-10 by mouse lymphoid cells and elevates its production after stimulation with Con A, but paradoxically also significantly stimulates production of TNF-α by peritoneal macrophages. Effects on IL-6 and IL-1β production were not significant.

Pseudhymenochirin-1Pb and pseudhymenochirin-2Pa were isolated from skin secretions of the frog *Pseudhymenochirus merlini* (Pipidae) [55], and both peptides are highly cytotoxic against human erythrocytes and several human tumour cell lines and potently inhibit the growth of a range of multidrug-resistant Gram-positive and Gram-negative bacteria [40]. Both pseudhymenochirin-1Pb and pseudhymenochirin-2Pa significantly inhibit the production of the anti-inflammatory cytokine IL-10 and the multifunctional cytokine IL-6, but enhance the production of the pro-inflammatory IL-23 by both unstimulated and LPS-stimulated murine macrophages [40].

Peptide glycine-leucine-amide (PGLa)-AM1, caerulein-precursor fragment (CPF)-AM1 and magainin-AM1 are cationic amphipathic α-helical peptides originally isolated from norepinephrine-stimulated skin secretions of the African volcano frog *Xenopus amieti* (Pipidae) [56] that exhibit relatively high growth-inhibitory activity against several oral and respiratory pathogens [41]. Production of the pro-inflammatory cytokine IL-8 by oral fibroblasts was significantly increased following treatment with magainin-AM1, but not by treatment with PGLa-AM1 or CPF-AM1.

Three structurally-related peptides belonging to the frenatin family (frenatin 2.1S, 2.2S and 2.3S) were isolated from skin secretions of the Orinoco lime tree frog *Sphaenorhynchus lacteus* (Hylidae) [33]. Frenatin 2.1S and 2.2S are *C*-terminally α-amidated glycine/leucine-rich peptides, while frenatin 2.3S lacks an amidated *C*-terminus. Frenatin 2.1S and 2.2S are active against methicillin-resistant *Staphylococcus aureus* (MRSA) and *Staphylococcus epidermidis* and are cytotoxic against non-small cell lung adenocarcinoma A549 cells. An immunostimulatory effect of frenatin 2.1S and 2.2S was documented by a significantly increased production of IL-1β and IL-23 by LPS-stimulated mouse peritoneal macrophages. In addition, frenatin 2.1S increased the production of proinflammatory TNF-α, while frenatin 2.2S decreased the production of anti-inflammatory IL-10 [33]. Immunostimulatory effects of frenatin 2.1S in vivo were demonstrated following intraperitoneal injection of 100 μg of the peptide into C57BL/6 and BALB/c mice. The peptide enhanced the immunostimulatory phenotype of peritoneal cells of C57BL/6 mice by increasing the expression of early activation marker CD69 among T and NKT cells and CXCR3 chemokine receptor on T cells, thereby indicating their activation status, as well as the polarization of the adaptive immune response toward the Th1 phenotype. Furthermore, frenatin 2.1S favours activation of peritoneal macrophages as evaluated by increased expression of MHC II, costimulatory molecule CD86 and mannose receptor CD206 among F4/80+CD11c+ cells [57].

Further studies demonstrated that intraperitoneal injection of 100 μg of frenatin 2.1S markedly increased the presence of peritoneal NK cells and promoted their activation status by increasing the expression of NK cells’ activation markers CD69, CD107a, NKG2D and FasL. Frenatin 2.1S enhanced the tumoricidal capacities of NK cells against the 4T1 mouse breast tumour cell line. Moreover, frenatin 2.1S promoted the presence of CD11c+ dendritic cells and CD3+ T lymphocytes in the peritoneal cavity of BALB/c mice and favoured the inflammatory phenotype of immune cells, as demonstrated by the increased presence of IL-17 and CXCR3-positive T lymphocytes and TNF-α-expressing macrophages [58]. The immunomodulatory activities of frenatin 2.1S are summarized in Table 3.

## 6. Conclusions

This article has provided evidence that several frog skin host defence peptides promote the activation of both innate and adaptive immunity as evaluated by cytokine production following in vitro challenge of macrophages and/or lymphoid cells. As well as their direct antimicrobial effects, such skin peptides may act as valuable stimulators of innate immunity in order to combat invading pathogens. Although frenatin 2D and plasticin-L1 did not show any direct antimicrobial activity, they are potent stimulators of macrophages as demonstrated by significantly increased production of proinflammatory cytokines [59]. Furthermore, several structurally-different frog skin peptides including esculentin-2CHa, pseudhymenochirins-1Pb and -2Pa, magainin-AM1, as well as frenatins 2.1S, 2.2S and 2.3S, exerted both antimicrobial and immunostimulatory activities, making them even more useful candidates for development into pharmaceutical agents.

The immunostimulatory effects of [G11k,N15K]alyteserin-2a, esculentin-2CHa and pseudhymenochirins 1Pb and 2Pa on peritoneal macrophages are complemented by the inhibition of anti-inflammatory IL-10 production, indicating a possible role in ameliorating the innate immune response during infection. In vivo studies have implicated frenatin 2.1S in promoting polarization of macrophages toward the proinflammatory M1 phenotype and T cells towards the Th1- and Th17-type of adaptive immune responses that are critically involved in eliminating extracellular and intracellular microorganisms [60,61]. On the other hand, the analogues [E6k,D9k]hymenochirin-1B, [D4k]ascaphin-8, [G4K]XT-7, [T5k]temporin-DRa, brevinin-2GU and B2RP-ERa kill bacteria directly, while enhancing the release of anti-inflammatory cytokines and/or suppressing the release of proinflammatory cytokines. These effects might be important both for eliminating the pathogen and providing the proper balance of immune response during infection, which might be a useful approach for sepsis therapy [62]. Future work should clarify whether immunoregulatory peptides may be useful in the cytokine storm during experimental sepsis.

Manipulation of the host immune system has long been a widely accepted approach for the treatment of various inflammatory and autoimmune diseases, as well as for suppression of tumours. IL-10 is established as a powerful suppressor of effector functions of macrophages, T cells and NK cells, including inhibition of Th1 cells, induction of regulatory T cells and production of proinflammatory cytokines and microbicidal compounds by macrophages [63,64]. Accordingly, there is a long-standing interest in exploiting the anti-inflammatory properties of IL-10 for immuno-intervention in autoimmune diseases [65]. Frog skin-derived tigerinins-1M, -1R and -1V and [E6k,D9k]hymenochirin-1B are potent stimulators of IL-10, whereas tigerinins-1O, -2O, -3O and -4O, rhinophrynin-27 and the analogues [D4k]ascaphin-8, [G4K]XT-7, [T5k]temporin-DRa, brevinin-2GU and B2RP-ERa suppress the production of proinflammatory cytokines contributing to the immunosuppressive properties of these peptides.

[G11k,N15K]alyteserin-2a, esculentin-2CHa, pseudhymenochirins 1Pb and 2Pa, magainin-AM1 and frenatins 2.1S, 2.2S and 2.3S are themselves directly cytotoxic for several human tumour cell lines, but their potential for anti-cancer activities may also involve their immunostimulatory properties [66]. The best studied peptide, frenatin 2.1S, is an enhancer of inflammatory phenotypes of mononuclear cells, including proinflammatory M1 macrophages, T and NKT cells. NK cells are crucial participants in the protection against infections and tumours mainly as a result of their powerful cytolytic machinery [67,68]. The most pronounced effect of frenatin 2.1S is accumulation of NK cells, as well as promotion of their activation and tumoricidal capacities following application in vivo.

Overall, it is clear that the frog skin host defence peptides, through the combined antimicrobial, cytotoxic and immunomodulatory activities, show potential for development into agents to treat infections, inflammatory and autoimmune diseases, as well as for anti-cancer therapy. Despite the fact that the magainin analogue, pexiganan, showed initial promise for treatment of infected foot ulcers in patients with diabetes [69], the potential of frog skin peptides as therapeutic agents has not been realized. No peptides based on their structures have yet been adopted in clinical practice either as anti-infective agents or as anti-inflammatory drugs. At this time, the promotion of wound healing appears to be the most promising application for frog skin peptides because of their abilities to prevent microbial invasion, reduce inflammation and induce migration of keratinocytes [70]. Several candidates have shown therapeutic relevance [28,71,72]. Obstacles to such applications include the cytotoxicity of many frog skin peptides for healthy human cells, as well as their short half-lives in circulation. Further investigations are needed to create long-acting analogues of the peptides that exhibit decreased cytolytic actions against human cells. Several strategies have evolved to increase the stability of peptide-based agents by increasing their resistance to degradation by peptidases. These include incorporation of D-amino acids or unnatural amino acids such as α-aminoisobutyric acid into the molecule and coupling of the peptide to a fatty acid such as palmitate or octanoate or to polyethylene glycol (reviewed in [73]). Furthermore, the mechanisms by which frog skin peptides exert their immunomodulatory actions are still obscure. Consequently, further studies are needed to elucidate the nature of the interaction of the peptide with the immune cell membrane and its effect on intracellular signaling pathways.

## Figures and Tables

**Table 1:** Species, common names and families of the frogs from which immunomodulatory peptides have been isolated.

Species	Common Name	Family
*Ascaphus truei*	Tailed frog	Ascaphidae
*Alytes obstetricans*	Midwife toad	Alytidae
*Discoglossus sardus*	Tyrrhenian painted frog	Alytidae
*Rhinophrynus dorsalis*	Mexican burrowing toad	Rhinophrynidae
*Hymenochirus boettgeri*	Congo dwarf clawed frog	Pipidae
*Pseudhymenochirus merlini*	Merlin’s clawed frog	Pipidae
*Xenopus tropicalis*	Tropical clawed frog	Pipidae
*Xenopus muelleri*	Mueller’s clawed frog	Pipidae
*Xenopus amieti*	Volcano clawed frog	Pipidae
*Hoplobatrachus rugulosus*	Vietnamese lowland frog	Dicroglossidae
*Hoplobatrachus occipitalis*	African crowned bullfrog	Dicroglossidae
*Leptodactylus laticeps*	Sante Fe frog	Leptodactylidae
*Sphaenorhynchus lacteus*	Orinoco lime frog	Hylidae
*Hylarana guentheri*	Guenther’s frog	Ranidae
*Hylarana erythraea*	Green Paddy frog	Ranidae
*Lithobates vaillanti*	Vaillant’s frog	Ranidae
*Lithobates chiricahuensis*	Chiricahua leopard frog	Ranidae
*Rana draytonii*	California red-legged frog	Ranidae

**Table 2:** Diverse effects of frog skin-derived peptides on cytokine production by human and murine cells.

Peptide	Primary Structure	Peritoneal Cells (PC) and/or Isolated Macrophages (M)	Spleen Cells (SC) and/or Human Peripheral Mononuclear Cells (hPMC)	Ref.
[D4k]ascaphin-8	GFKkLLKGAAKALVKT VLF ^a^	n.d.	TNF-α↓, IFN-γ↓ (hPMC)	[30]
Alyteserin-2a	ILGKLLSTAAGLLSNL ^a^	n.d.	IL-10↓, TGF-β↓ (hPMC)	[31]
Frenatin-2D	DLLGTLGNLPLPFI ^a^	TNF-α↑, IL-1β↑, IL-12↑ (M)	n.d.	[32]
Frenatin 2.1S Frenatin 2.2S	GLVGTLLGHIGKAILG ^a^ GLVGTLLGHIGKAILS ^a^	TNF-α↑, IL-1β↑, IL-23↑ (M) IL-1β↑, IL-23↑, IL-10↓ (M)	n.d.	[33]
Plasticin-L1	GLVNGLLSSVLGGGQGGGGLLGGIL	TNF-α↑, IL-1β↑, IL-12↑, IL-23↑, IL-6↑ (M)	n.d.	[34]
Tigerinin-1R	RVCSAIPLPICH ^a^	IL-10↑, IL-6↑ (M)	IL-10↑ (SC, hPMC)	[35]
Tigerinin-1V	RICYAMWIPYPC	IL-10↑, IL-6↑ (M)	IL-10↑ (SC, hPMC), IFN-γ↓ (SC)	[35]
Tigerinin-1M	WCPPMIPLCSRF ^a^	IL-10↑ (M), IL-6↑ (M)	IL-10↑ (SC, hPMC), IFN-γ↓ (SC)	[35]
Tigerinin-1O Tigerinin-2O Tigerinin-3O Tigerinin-4O	RICTPIPFPMCY RTCIPIPLVMC RICTAIPLPMCL RTCIPIPPVCF	IFN-γ↓ (PC)	n.d.	[36]
Rhinophrynin-27	ELRLPEIARPVPEVLPARLPLPALPRN	TNF-α↓ (M)	n.d.	[37,38]
[E6k,D9k] hymenochirin-1B	IKLSPETKDNLKKVLKGAI AVAKMV ^a^	n.d.	IL-10↑, IL-4↑ (hPMC)	[39]
Pseudhymenochirin-1Pb Pseudhymenochirin-2Pa	IKIPSFFRNILKKVGKEAVSLIAGALKQS GIFPIFAKLLGKVIKVASSLISKGRTE	IL-10↓, IL-6↓, IL-23↑ (M)	n.d.	[40]
[G4K]XT-7	GLLKPLLKIAAKVGSN LL ^a^	n.d.	TNF-α↓ (hPMC)	[30]
Magainin-AM1	GIKEFAHSLGKFGKAFVGGILNQ	IL-8↑ (oral fibroblasts)	n.d.	[41]
Brevinin-2GU	GVIIDTLKGAAKTVAAELLRKAHCKLTNSC	n.d.	TNF-α↓, IFN-γ↓ (hPMC)	[30]
B2RP-ERa	GVIKSVLKGVAKTVAL GML ^a^	n.d.	TNF-α↓, TGF-β↑, IL-4↑, IL-10↑ (hPMC)	[30]
[T5k]temporin-DRa	HFLGkLVNLAKKIL ^a^	n.d.	TNF-α↓, TGF-β↑, IL-4↑, IL-10↑ (hPMC)	[30]
Esculentin-2CHa	GFSSIFRGVAKFASKGLGKDLAKLGVDLVACKISKQC	TNF-α↑ (M)	IL-10↑ (SC)	[42]

n.d., not determined; ^a^ denotes *C*-terminal α-amidation.

**Table 3:** In vivo effects of the frog skin-derived peptide frenatin 2.1S on murine peritoneal mononuclear cells.

Immune Cells	Effects of Frenatin 2.1S Administration	Ref.
NK cells	Increased presence of CD3-CD49b+ NK cells Activation of NK cells as evaluated by increased expression of CD69, CD107a, NKG2D, FasL	[58]
T cells	Increased presence of CD3+ T cells Activation of T cells as evaluated by increased expression of: chemokine receptor CXCR3 IL-17 CD69	[57,58]
NKT cells	Activation of NKT cells as evaluated by increased expression of CD69	[57]
Macrophages	Macrophage activation as evaluated by increased expression of: MHC II mannose receptor CD206 costimulatory molecule CD86 TNF-α	[57,58]
Dendritic cells	Increased presence of CD11c+ cells	[57,58]
